# Primary and secondary hyperparathyroidism present different expressions of calcium-sensing receptor

**DOI:** 10.1186/s12893-023-01928-5

**Published:** 2023-02-08

**Authors:** Xin Li, Yao Lu, Ling Zhang, Aiping Song, Honglei Zhang, Bo Pang, Jun Liu, Xiaoliang Sun, Haoyang Ji, Linping Huang, Meng Yang

**Affiliations:** 1 Institute of Clinical Medicine Research, China-Japan Friendship Hospital, Beijing, 100029 China; 2 Department of General Surgery, China-Japan Friendship Hospital, Beijing, 100029 China; 3 Center of Nephrology, China-Japan Friendship Hospital, Beijing, 100029 China; 4 Department of Pathology, China-Japan Friendship Hospital, Beijing, 100029 China; 5 State Key Laboratory of Infectious Disease Prevention and Control, National Institute for Communicable Disease Control and Prevention, China CDC 155, Changbai Road, Changping, Beijing, 102206 China

**Keywords:** Calcium-sensing receptor, Secondary hyperparathyroidism, Primary hyperparathyroidism

## Abstract

**Background:**

Decreased calcium-sensing receptor (CaSR) has been observed in hyperparathyroidism (HPT) without a known mechanism. The purpose of this study was to evaluate the expression of CaSR in primary (PHPT) and secondary (SHPT) subtypes.

**Methods:**

Immunohistochemical (IHC) staining and quantitative real-time PCR (qRT-PCR) assay were used to measure the differences in expression of CaSR protein and gene in PHPT and SHPT human samples, compared to matched controls.

**Results:**

CaSR protein was differentially downregulated in SHPT and PHPT compared to normal parathyroid tissues (2.42 ± 0.5 vs. 3.2 ± 0.62, P < 0.05; 1.8 ± 0.83 vs. 3.2 ± 0.62, P < 0.05, respectively). Furthermore, SHPT tissues exhibited significantly higher levels of CaSR mRNA (0.29 ± 0.23 vs. 0.01 ± 0.12, P < 0.05) and protein (2.42 ± 0.5 vs. 1.8 ± 0.83, P < 0.05) than those in PHPT tissue samples.

**Conclusion:**

Depressed CaSR expression was a critical pathological hallmark of HPT. We found a differential decline of CaSR, in terms of both mRNA and protein levels, in PHPT and SHPT human samples. We think that CaSR dysregulation occurred at the very beginning of disease onset in PHPT, while a similar pathological scenario appeared at the later stage of SHPT. Future studies should be directed to dissect the mechanistic involvement of CaSR in PHPT and SHPT in order to bring treatment precisions in HPT management.

## Background

Hyperparathyroidism (HPT) is characterized by abnormally increased production of parathyroid hormone (PTH) and the resulting disordered calcium and phosphorus metabolism. Clinically HPT can be categorized as the primary (PHPT) and secondary (SHPT) subtypes. PHPT is caused by the disorder in one of the parathyroid glands itself producing an excessively high level of PTH, while SHPT is related to the abnormally overactive behavior of the parathyroid glands secondary to the underlying disease [1]. Parathyroid adenoma (PTA) is thought to be the most common cause of PHPT. The sporadic form of PTA has been frequently reported in 80–90% of PHPT cases, and familial form of the pathology contributes rest 10–20% of cases. It has been shown that genetically inherited mutations in the multiple endocrine neoplasia type 1 (*MEN1*) and Ret Proto-Oncogene (*RET*) are linked to the development of endocrine tumors [2]. However, the etiopathological factors of sporadic PHPT have not been explored in detail. Clinical investigations have implicated the role of germline or somatic mutation in the calcium-sensing receptors (CaSRs) as the causal factor in PHPT onset [3]. Koh et al. [4] found that CaSR is one of the genes dysregulated in parathyroid adenomas and mediating parathyroid biology.

SHPT is a common disorder that occurs in chronic kidney disease (CKD), characterized by significantly reduced expressions of CaSR and vitamin D receptor (VDR) [5], which largely contributes to death and cardiovascular events in CKD patients. CaSR and VDR-mediated signaling have been linked to the PTH gene activation through transcriptional regulation and subsequent proliferation of parathyroid glands. Pathological examinations of parathyroid glands in patients with chronic CKD and/or end-stage renal diseases (ESRD) have revealed transformation from diffused to nodular hyperplasia [5], accompanied by the decrease in CaSR and VDR levels. However, the underlying mechanisms remain poorly understood.

Both PHPT and SHPT have been pathologically linked to reduced CaSR expression and altered bone mineral density in general, but the specific involvement of proteins and genes in PHPT and SHPT pathomechanism has not been explored so far [3, 4]. Few studies have compared CaSR expression in PHPT and SHPT.Therefore, we aimed to investigate differential protein and gene expression related to HPT subtypes using human tissue samples in this study.

## Methods

### Patient selection

Parathyroid tissue samples were collected from patients who had confirmed pathological diagnosis of either PHPT or SHPT and underwent surgery at the China-Japan Friendship Hospital between 2013 and 2016. Control samples were obtained from a tissue biorepository of the hospital, which was collected during the accidental removal of the parathyroid gland from patients who had the non-HPT disease during thyroidectomy.

### Immunohistochemistry

Immunohistochemical (IHC) staining against CaSR was performed to pathologically distinguish between PTA, nodular hyperplasia, and normal parathyroid gland tissue samples. In total, 71 formalin-fixed paraffin-embedded (FFPE) tissues, including 20 PHPT, 31 SHPT, and 20 controls, were examined by IHC. From the FFPE tissue blocks, 4 µm-thick sections were prepared using a slicer, and respective tissue sections were probed with mouse anti-CaSR monoclonal antibody (Thermo Fisher, MA1-934) in 1:1500 dilution by EnVision/HRP method using an automatic immunostainer. Microscopic evaluations of tissue sections were performed under 20 × and 40 × optical resolutions. A total of 500 cells (100 cells per region) were counted to determine the relative fold change of CaSR expression with respect to matched controls. To keep the evaluation consistent across the experiments, we set up a scoring grade for CaSR expression level, like + (1 +) indicated week expression, +  + (2 +)as moderate, +  +  + (3 +) as strong, and +  +  +  + (4 +) referred to a very strong expression level. Observers were kept blind to patient details related to individual samples until analyses were complete.

### RNA isolation and quantitative real-time PCR (qRT-PCR) assay

All tissue samples were immediately stored at − 80 °C followed by RNA isolation. Total RNA was extracted using the TaKaRa MiniBEST universal RNA extraction kit (TaKaRa Biochemicals, Japan). RNA quantity and quality were tested on a NanoDrop instrument prior to cDNA synthesis. The first-strand cDNA synthesis was performed using the PirmeScript™ 1st-strand cDNA synthesis kit (TaKaRa). Reactions were prepared with SYBR Premix Ex Taq™ (TaKaRa) and subsequently ran on the ABI 7500 FAST RT-PCR detection system (ABI, USA). The primer sequences used to measure the expressions of CaSR and β-actin as internal loading control in this qRT-PCR assay are shown in Table 1. The specificity of the PCR reaction was evaluated by melting curve analysis. The relative expression of CaSR across the samples was measured by 2^−∆Ct^ method.

**Table 1:** Primers used for realtimePCR

Gene	Forward primer	Reverse primer
CASR [6]	CGGGGTACCTTAAGCACCTACGGCATCTAA	GCTCTAGAGTTAACGCGATCCCAAAGGGCTC
β-actin [4]	ACTCTTCCAGCCTTCCTTCC	CAGGAGGAGCAATGATCTTG

### Statistical analysis

Data were subjected to statistical analysis using the SPSS software. Quantitative data between multiple groups were analyzed by analysis of variance (ANOVA), and between two groups were analyzed by independent sample *t*-test for parametric test. Values were expressed as mean ± standard deviation (SD). P ≤ 0.05 was considered statistically significant.

## Results

Here, we compared the relative expression of CaSR in 71 tissue samples, including 20 PHPT, 31 SHPT, and 20 controls, by IHC analysis. For patients with PHPT, mean pre-operative serum levels were 799.5 ± 995.35 pg/mL for PTH, 3.12 ± 0.5 mmol/L for total calcium, 0.77 ± 0.21 mmol/L for P, and 156.75 ± 119.13 U/L for ALP. For patients with SHPT, mean pre-operative serum levels were 2053.84 ± 822.29 pg/mL for PTH, 2.59 ± 0.25 mmol/L for total calcium, 2.29 ± 0.43 mmol/L for P, and 434.32 ± 346.21 U/L for ALP. 75% of PHPT patients have clinical symptoms, including low back pain, leg pain, urinary calculi and loss of appetite, etc. 20% of PHPT patients have renal impairment.

CaSR is generally stained in the cell membranes and cytoplasm of SHPT and PHPT patients. The staining intensity of CaSR in SHPT patients is significantly higher than that in PHPT patients, and there is no significant difference in staining sites.

**Fig. 1:**
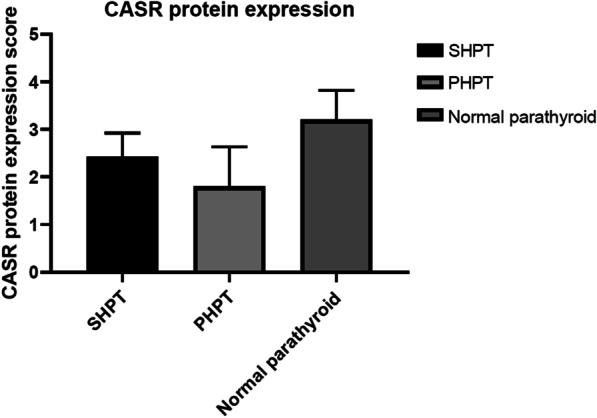
ANOVA test for CaSR protein expression in different parathyroid tissues, P < 0.05

**Fig. 2:**
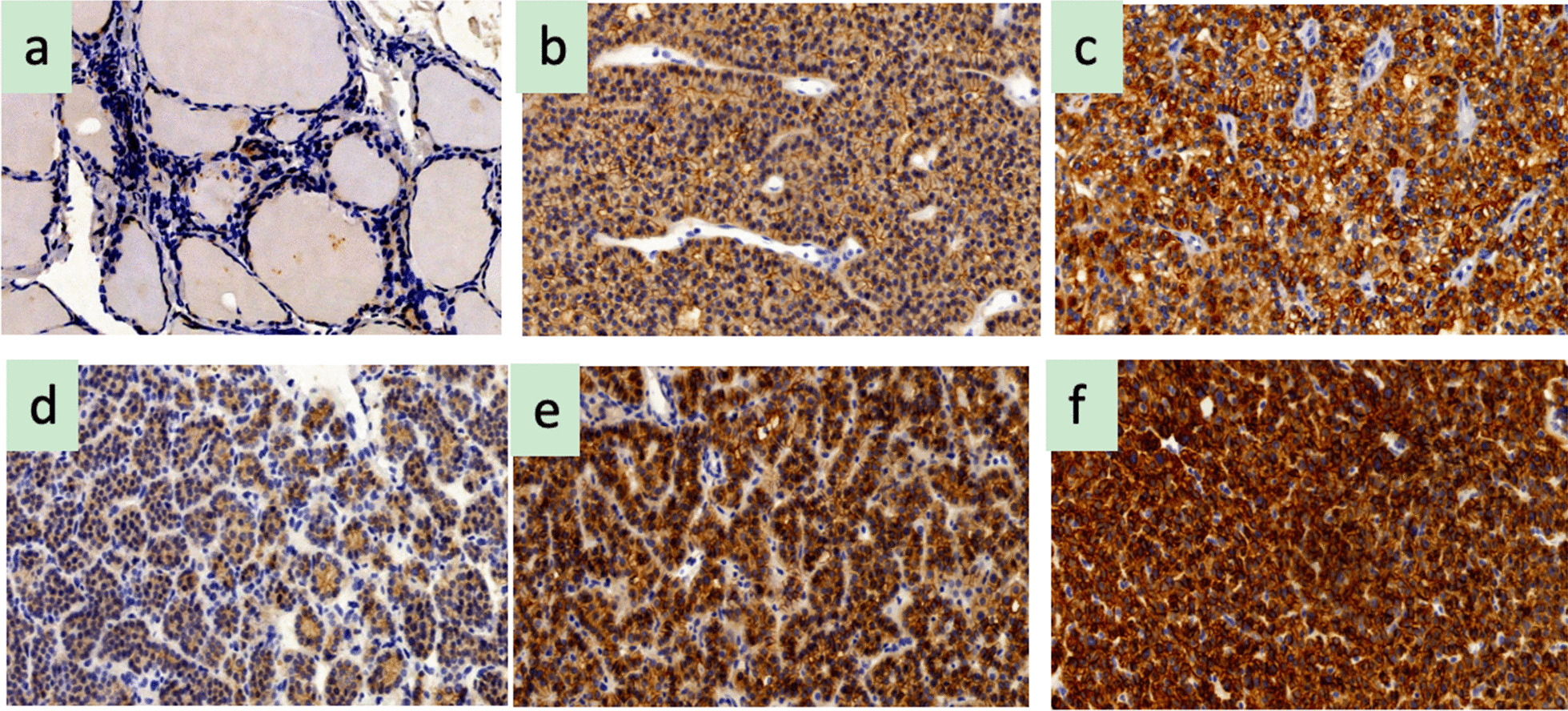
Immunohistochemical staining of CaSR. **a** Thyriod glands (0, × 20); **b** secondary hyperparathyroidism (+ + , × 20); **c** secondary hyperparathyroidism tissue (+ +  + , × 20); **d** primary hyperparathyroidism tissue (+ , × 20);); **e** primary hyperparathyroidism tissue (+ + , × 20);); **f** primary hyperparathyroidism tissue (+ +  + , × 20);

**Fig. 3:**
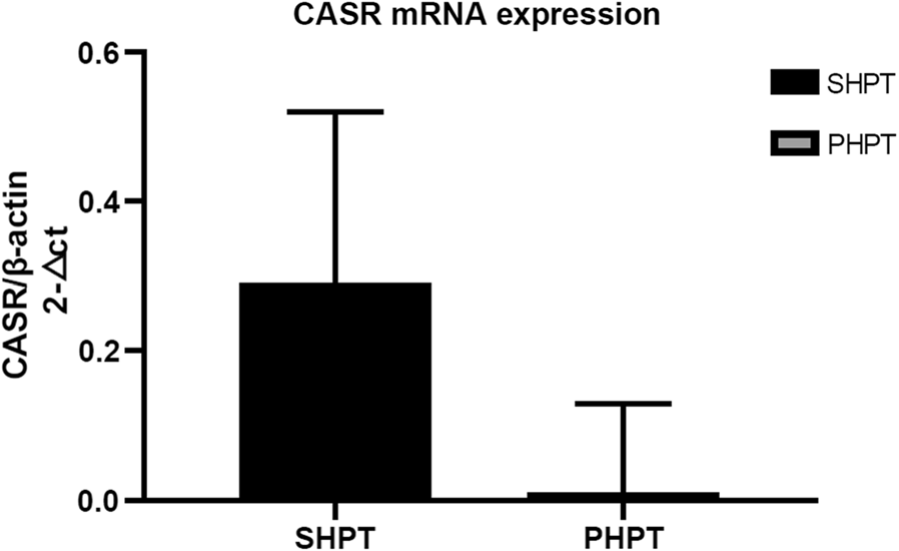
T test for SHPT and PHPT CaSR gene expression (real time PCR 2^−∆ct^), P < 0.05

We found that PHPT was associated with the lowest (1.8 ± 0.83) level of CaSR, followed by the SHPT group (2.42 ± 0.5) compared to that of the control group (3.2 ± 0.62). In other words, CaSR protein level was strongly impacted in PHPT than in the SHPT pathology (2.42 ± 0.5 vs. 1.8 ± 0.83, P < 0.05), while the CaSR level in the SHPT group was significantly lower than that of the control group (2.42 ± 0.5 vs. 3.2 ± 0.62, P < 0.05) (Table 2, Figs. 1, 2). We could not detect any expression variation of CaSR in the thyroid tissues (Fig. 2). The qRT-PCR-based quantitation of CaSR mRNA levels was performed in 47 HPT cases (31 SHPT and 16 PHPT), which revealed that the expression of CaSR mRNA in SHPT was higher than that in PHPT (0.29 ± 0.23 vs. 0.01 ± 0.12, P < 0.05) (Table 3 and Fig. 3).

**Table 2:** ANOVA test for CaSR protein expression in different parathyroid tissues

	Quantity	Expression value mean ± SD	P value
SHPT	31	2.42 ± 0.5	< 0.05
PHPT	20	1.8 ± 0.83	< 0.05
Normal parathyroid	20	3.2 ± 0.62	< 0.05

**Table 3:** T test for CaSR mRNA expression (realtimePCR 2^−∆ct^)

	Quantity	CASR/β-actin 2^−∆ct^ (‾x ± sd)	P value
SHPT	31	0.29 ± 0.23	< 0.05
PHPT	16	0.01 ± 0.12	< 0.05

## Discussion

The human CaSR gene is located on chromosome 3q13.3-21 and is abundantly expressed in the parathyroid gland, kidney, and parafollicular cells or C cells near the thyroid follicles. CaSR, a G protein-coupled receptor (GPCR), is the key factor in the cellular calcium sensing mechanism and maintaining calcium homeostasis in a cell type-specific manner. Under normal physiological conditions, calcium-activated CaSR triggers the mitogen-activated protein kinase C (MAPK) signaling cascade inducing the synthesis of phospholipase A2 (PLA2), which in turn catalyzes the hydrolysis of the membrane glycerophospholipids to generate arachidonic acid (AA), resulting in the decreased synthesis and secretion of PTH [7]. Thus, CaSR activation can lead to inhibition of the parathyroid cell proliferation, synthesis of 1,25-dihydroxy vitamin D3, and reduction in the renal calcium reabsorption in the distal tubules.

Our results showed that HPT, irrespective of its subtypes, significantly downregulated CaSR expression compared to that of the control parathyroid tissue samples. Furthermore, a direct disorder of the parathyroid glands, including PTA-related pathology, in PHPT showed a profound effect on the CaSR expression both at the mRNA and protein levels, as compared to that of the SHPT. Although several studies have confirmed that CaSR downregulation in SHPT is a critical pathological hallmark and driving force in disease progression, the underlying mechanisms have not yet been delineated. Brown et al. [8] have revealed that suppression of CaSR expression in the nodular region is quite common in the rat model of SHPT. Another study also showed that CaSR expression was significantly reduced in the parathyroid area in the uremic rat model [9]. Yano et al. [10] also confirmed this observation in proliferative human parathyroid tissue. Ritter et al. [11] suggested that a decrease in the CaSR level could be the effect of the onset of hyperparathyroidism, possibly due to the parathyroid cell proliferation.

Likewise, a high level of CaSR expression reduction was also observed in PHPT, however, most likely due to inherited or acquired genetic mutation-associated aberrant stimulation of parathyroid glands [12]. Cyclin D1 (PRAD1) oncogene rearrangement and its transcriptional activation have been implicated in the tumorigenesis of PTA [13]. Deficient calcium sensing and disrupted feedback to PTH occur early in the pathogenic process of parathyroid cells’ hyperplastic expansion [14]. Koh et al. have shown that abnormal upregulation of the Regulator Of G Protein Signaling 5 (*RGS5*) gene can influence CaSR signaling in the PHPT [4, 15]. Furthermore, vitro study has demonstrated that the Glial Cells Missing Transcription Factor 2 (GCM2) protein regulates the CaSR expression [16]. In PHPT, impaired calcium sensing results in elevated PTH secretion and parathyroid neoplasia [15].

Differences in the extent of CaSR reduction in PHPT and SHPT compared to healthy control suggest an involvement of diverse pathomechanisms. Studies on the SHPT mechanism indicate that long-term uremic dialysis causes increased phosphate retention, leading to hyperphosphatemia followed by hypocalcemia. Up to this point, the CaSR level maintains at a normal physiological level; hence, parathyroid hyperplasia gets stimulated with accelerated PTH secretion. During this process, CaSR expression decreases, inducing a vicious cycle of repeated PTH secretion enhancement in a feedback loop. In order to adapt to the persistently increased PTH level, parathyroid cells undergo uncontrolled proliferation developing PTA and hypercalcemia. However, in PHPT due to depressed CaSR expression onset, the feedback mechanism between calcium and PTH is disrupted so that hypercalcemia fails to block PTH secretion, thereby aggravating the PHPT symptoms**.** These mechanisms can partly explain the observed differences in CaSR decline between PHPT and SHPT, despite certain overlapping mechanisms in these two diseases. Notably, the advanced stage of SHPT onsets tertiary HPT activating parathyroid glands for autonomous secretion of PTH. At this stage, nodular hyperplasia is pathologically very similar to PHPT.

In the view of available knowledge resources on HPT-associated calcium sensing deficits, our study with human samples provided the foundation for future investigations to explore the exact mechanisms of CaSR dysregulation in SHPT and PHPT to benefit the treatment of HPT.

1. There are few studies on the difference of CaSR expression between PHPT and SHPT. Our advantage is to explore the clues of the pathogenesis of hyperparathyroidism by studying the expression difference of CaSR in hyperparathyroidism. Our study also had some limitations, such as a small sample size, lack of long-term follow-up data, and lack of multicenter data. Because normal parathyroid gland is very difficult to obtain, it was not included in the CaSR mRNA expression.

## Conclusion

Depressed CaSR expression was a critical pathological hallmark of HPT. We found a differential decline of CaSR, in terms of both mRNA and protein levels, in PHPT and SHPT human samples. We think that CaSR dysregulation occurred at the very beginning of disease onset in PHPT, while a similar pathological scenario appeared at the later stage of SHPT. Future studies should be directed to dissect the mechanistic involvement of CaSR in PHPT and SHPT in order to bring treatment precisions in HPT management.

## Data Availability

The data that support the findings of this study are available on request from the corresponding author. The data are not publicly available due to privacy or ethical restrictions.

## References

[1] Dandurand K, Ali DS, Khan AA. Primary hyperparathyroidism: a narrative review of diagnosis and medical management. J Clin Med. 2021; 10(8). 10.3390/jcm10081604 PMC8068862 33918966

[2] Heppner C (1997). Somatic mutation of the MEN1 gene in parathyroid tumours. Nat Genet.

[3] Sengul AG (2018). Clinical Impact of p27(Kip1) and CaSR expression on primary hyperparathyroidism. Endocr Pathol.

[4] Koh J (2011). Regulator of G protein signaling 5 is highly expressed in parathyroid tumors and inhibits signaling by the calcium-sensing receptor. Mol Endocrinol.

[5] Uchiyama T (2016). Hypermethylation of the CaSR and VDR genes in the parathyroid glands in chronic kidney disease rats with high-phosphate diet. Hum Cell.

[6] Sanders JL (2000). Extracellular calcium-sensing receptor expression and its potential role in regulating parathyroid hormone-related peptide secretion in human breast cancer cell lines. Endocrinology.

[7] Brennan SC, Conigrave AD (2009). Regulation of cellular signal transduction pathways by the extracellular calcium-sensing receptor. Curr Pharm Biotechnol.

[8] Brown AJ (1999). Decreased calcium-sensing receptor expression in hyperplastic parathyroid glands of uremic rats: role of dietary phosphate. Kidney Int.

[9] Ritter CS (2002). Reversal of secondary hyperparathyroidism by phosphate restriction restores parathyroid calcium-sensing receptor expression and function. J Bone Miner Res.

[10] Yano S (2000). Association of decreased calcium-sensing receptor expression with proliferation of parathyroid cells in secondary hyperparathyroidism. Kidney Int.

[11] Ritter CS (2001). Parathyroid hyperplasia in uremic rats precedes down-regulation of the calcium receptor. Kidney Int.

[12] Corbetta S (2000). Calcium-sensing receptor expression and signalling in human parathyroid adenomas and primary hyperplasia. Clin Endocrinol (Oxf).

[13] Motokura T (1991). A novel cyclin encoded by a bcl1-linked candidate oncogene. Nature.

[14] Imanishi Y (2011). Cinacalcet HCl suppresses Cyclin D1 oncogene-derived parathyroid cell proliferation in a murine model for primary hyperparathyroidism. Calcif Tissue Int.

[15] Balenga N (2019). Parathyroid-targeted overexpression of regulator of G-protein signaling 5 (RGS5) causes hyperparathyroidism in transgenic mice. J Bone Miner Res.

[16] Mizobuchi M (2009). Calcium-sensing receptor expression is regulated by glial cells missing-2 in human parathyroid cells. J Bone Miner Res.

